# Risk‐based Decision Making Definition: A Scoping Review of Food, Agricultural, Environmental, and Medical Literature

**DOI:** 10.1111/risa.13845

**Published:** 2021-10-27

**Authors:** Kara M. Morgan, Ashley Crawford, Barbara B. Kowalcyk

**Affiliations:** ^1^ Center for Foodborne Illness Research and Prevention, and Department of Food Science and Technology The Ohio State University Columbus OH USA; ^2^ College of Public Health The Ohio State University Columbus OH USA; ^3^ Translational Data Analytics Institute The Ohio State University Columbus OH USA

**Keywords:** Decision making, food safety, risk‐based

## Abstract

Risk‐based decision making (RBDM) is a term that is used frequently as an aspirational goal in many fields, including health, engineering, environmental science, regulatory and, more recently, food safety. When RBDM is used in the literature, many different types of criteria are used to characterize a decision process as being “risk‐based.” Like the parable about the blind men and the elephant, everyone is confident they know what RBDM means even though there is no universal definition. The use of RBDM is gaining wide acceptance and implies a level of rigor and focus that many decisionmakers and stakeholders are interested in adopting. However, without one clear definition, there are questions about what a RBDM approach really means. This study summarizes peer‐reviewed and gray literature that uses the term “RBDM” from the last 50 years in the agricultural, environmental, and medical areas. The criteria discussed were identified and organized into themes. A foundational definition is proposed to represent the most fundamental use of RBDM in the literature, and three themes covering the additional concepts presented in some of the literature were identified and added as themes within the definition. Results from this research will inform practitioners interested in following the principles of RBDM, and will help guide researchers who are interested in advancing this approach. The most immediate use will be to guide the development of a roadmap for a risk‐based food safety system for low‐ and middle‐income countries and to aid the global food safety community in moving toward RBDM.

## 1 INTRODUCTION

Risk‐based decision making (RBDM) is a term that is frequently used in a wide range of domains from engineering to medicine to environmental science. In food safety, risk‐based food safety systems are internationally recognized as the best approach for improving food safety but the use of RBDM is limited. Notably, RBDM does not have one clear definition despite its broad use. Rather, the multiple definitions offered across the peer‐reviewed and gray literature has created a challenge for those who are seeking to use an RBDM approach and is a limitation for those who aim to promote its use. A report by Organization for Economic Cooperation and Development on risk‐based regulatory policy (OECD, 2010) claims that labeling an analysis or decision as “risk‐based’ provides a level of gravitas and legitimacy. Without clarity about what RBDM means, that legitimacy could be unfounded.

For several decades, experts in many fields have advocated for the use of RBDM as the best way to manage risks. For example, in 1999, Peterman and Anderson highlighted the role of RBDM in natural resource management and, in particular, the need to address uncertainty when undertaking RBDM (Peterman & Anderson, 1999). One decade later, in 2010, a National Academies report on the role of the U.S. Food and Drug Administration on ensuring food safety made the argument that a risk‐based food safety system is a more effective system (Institute of Medicine and National Research Council, 2010). In 2011, a consensus conference published several papers regarding the use of RBDM for blood safety to address blood shortages (Leach Bennett, Blajchman, Delage, Fearon, & Devine, 2011; Stein et al., 2011). In 2014, a National Research Council Committee published proceedings from a workshop on “Best Practices for Risk‐Informed Decision Making Regarding Contaminated Sites” emphasized the need to include other factors in addition to public health risk (Committee on Best Practices for Risk‐Informed Remedy Selection, Closure and Post‐Closure of Contaminated Sites, Nuclear and Radiation Studies Board, Division on Earth and Life Studies, Science and Technology for Sustainability Program, Policy and Global Affairs, & National Research Council, 2014).

As noted above, there have been calls for using RBDM in food safety settings but what this approach looks like in practice has not been clearly defined. Recently, the World Health Organization published the first‐ever estimates of the global burden of foodborne disease (Havelaar et al., 2015). These estimates have increased awareness about the scope and impact of foodborne disease, particularly in low‐ and middle‐income countries where food safety has not been previously prioritized. While many countries recognize the importance of utilizing RBDM to allocate their limited food safety resources, many struggle with how to implement such an approach. Recognizing this, the Food and Agriculture Organization of the United Nations has recently issued several guidance documents related to RBDM (FAO, 2017, 2020), but the details of how to implement RBDM are still not clear. Specifically: What *is* a risk‐based food safety system? What are the elements that must be included to have a risk‐based food safety system? How do you know you have a risk‐based system? These questions are the underlying drivers for this research.

The objective of this study is to develop a definition for RBDM that can be implemented in a practical way across a broad range of domains and, particularly, in food safety. Having a clear definition will aid analysts and decisionmakers who are interested in using a RBDM approach. To make it implementable, the definition should include a description of the needed components so that progress toward establishing and improving RBDM systems can be assessed.

## 2 METHODS

A scoping review was undertaken to generate a description of how RBDM has been used in the existing literature. Scoping reviews are often used as initial steps to inform a more focused systematic review (Munn et al., 2018). In contrast to systematic reviews, a scoping review does not include a critical review of study design or an assessment of the quality of the study of the publications to be included (Peters et al., 2015). There was an expectation that identified literature would come from a variety of sources, since RBDM is used across a wide range of application areas. Due to the focus on food safety, the search focused on agricultural, environmental, and public health sources.

Search results were compiled from CAB Abstracts, Agricola, Food Science Source, Food Science and Technology Abstracts, and PubMed. To be included in this scoping review, a publication had to meet the following inclusion criteria: (1) used the term “risk‐based” to describe an act of decision making; (2) included explicit discussion of decision making, not just risk assessment; (3) discussed the meaning of RBDM (in some form) OR provided an illustrative case study that demonstrates the meaning with identified criteria, and (4) focused on human or environmental health risks. Publications were excluded if they focused on other types of risks such as project management risks or supply chain risks; if they were not accessible as full text to the researchers; or if they were not available in English.

The search strategy was designed to identify all publications that met the criteria. An initial set of search terms were developed to include variations of key concepts in the research question, including risk, governance, RBDM, risk‐based, decision making and multicriteria decision making. After the initial search returned an overwhelming number of publications, the search strategy was modified so that only publications that included at least two of these search terms were selected. Publications were included in this review if they included any of the three pairs of search terms: (1) “multicriteria decision making” AND “risk”; (2) “risk‐based” AND “governance”; and (3) “risk‐based” AND “decision making.”

Identified publications were screened and reviewed. The title and abstract of each publication were screened by one reviewer for relevance against the inclusion criteria, and publications that clearly did not meet the criteria were classified as not relevant. For publications deemed relevant, a full‐text review was conducted against the inclusion criteria by the same reviewer. Publications that met the inclusion/exclusion criteria underwent a full‐text review by a second reviewer. To ensure data quality, 15% of publications that were classified as not relevant by the first reviewer were randomly selected for review by the second reviewer and reassessed for relevance and eligibility for inclusion. Seven percent of the publications initially classified as not relevant were ultimately included in the scoping review. Reference management software (Zotero) was used for data management and to store information from the review. Publications included in the scoping review were analyzed in terms of their year of publication and their topic area. The topic categories used for this analysis (environmental science, engineering and infrastructure, medical, regulatory, food safety, agriculture, natural disasters, security and defense, international development, ecological, and public health) were selected in consultation with leadership from the Society for Risk Analysis, and for the most part are the categories that are used for planning the sessions at their annual meeting. The categories were assigned to each publication by the initial reviewer and were confirmed by the second reviewer.

## 3 RESULTS

### 3.1 Summary of Relevant Publications

**Fig 1:**
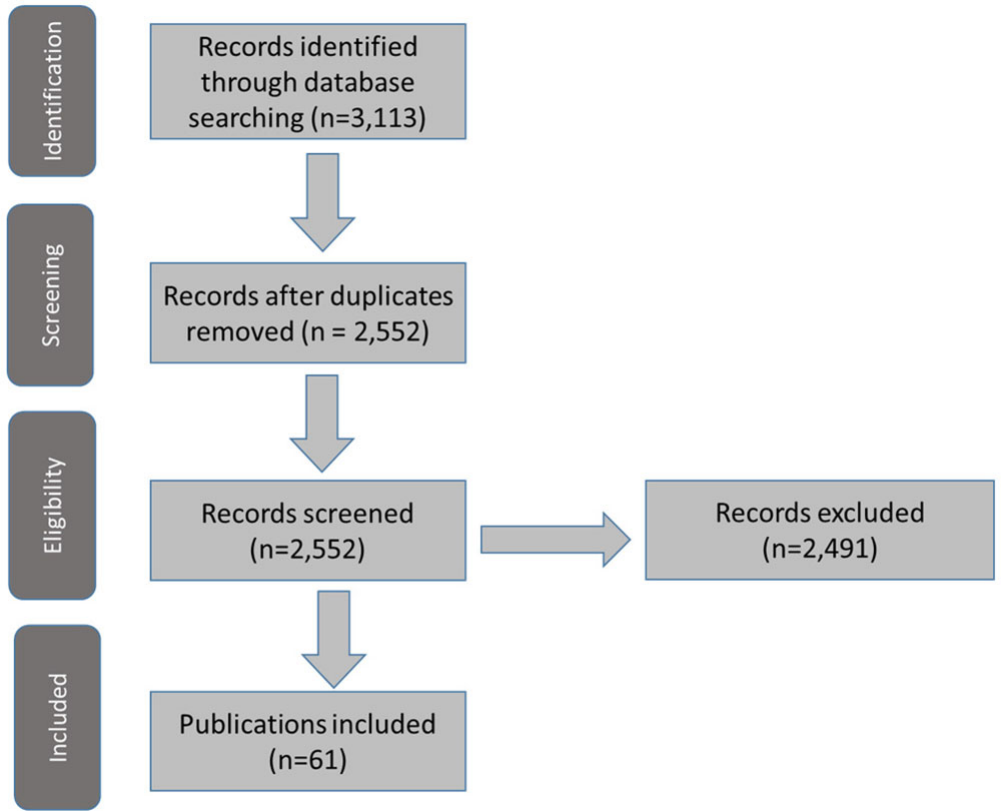
Scoping review results

This scoping review identified 2,552 unique publications. Of these, 61 (2%) met the inclusion/exclusion criteria and were included in the review (Fig. 1, Appendix A). Of these, 34 (56%) publications explicitly used the term “risk‐based decision making” and two (3%) used the term “risk‐informed.” The remaining 25 (41%) publications used “risk‐based” as a descriptor for the type of decision making they were doing, such as “risk‐based permitting,” “risk‐based corrective action,” or “risk‐based screening.”

**Fig 2:**
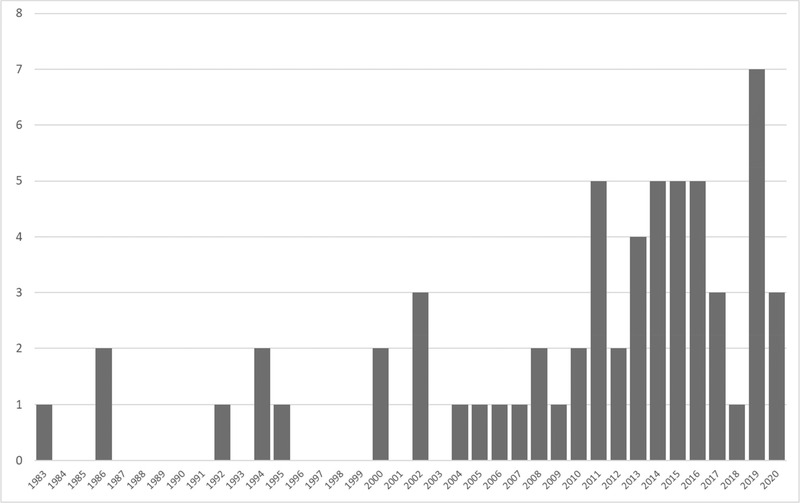
Timeline of included publications

**Fig 3:**
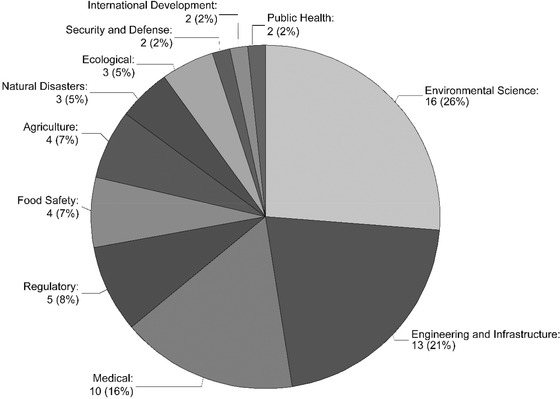
Topic areas in RBDM literature included in the scoping review

The number of RBDM publications has increased over time (Fig. 2). The first publication that met the stated criteria was published in 1983. Publication proceeded slowly after that, with increasing frequency in 2000 and a jump in annual contributions in 2011. The year with the most publications was 2019, with eight publications published. It should be noted that this review was completed in August 2020, so all relevant publications from 2020 may not have been identified. RBDM publications were found across 11 different topic categories with the largest percentages in environmental science (26%), engineering and infrastructure (21%), and medical (16%) (Fig. 3). The list of publications and the topic category they were assigned to can be found in Appendix A.

Environmental science, engineering and infrastructure, and medical were the topic areas with the most publications and the earliest publications, with the literature spanning 28, 33, and 36 years, respectively. Food safety, the driver for this research, had four publications with two of them being National Academy reports. They spanned the years 2010–2016. Agriculture had four papers with the first paper being published in 2000 and the most recent in 2019. Regulatory had five papers with the first being published in 2002 and the most recent in 2013. Ecological had only three papers with the first being published in 2006 and the next two in 2014 and 2015. The first papers in the other topics were all published since 2010.

Included publications were published in 34 different journals, indicating a lack of a central resource. *Science of the Total Environment* and *Risk Analysis* had the most publications with five and four, respectively. Eight journals had two or three publications, and 24 journals only had one publication. In addition to the journal publications, this scoping review identified two books or book chapters, two publications from conference proceedings, three publications from workshop proceedings, and three reports.

### 3.2 Foundational Characteristics of RBDM

Publications identified through this scoping review cover a wide breadth of applications and there were many variations in their definitions, as discussed below. In spite of this wide range, all of them contained two common characteristics. First, all publications included qualitative or quantitative estimates of likelihood and severity of harm (i.e., risk). Second, in each of these publications, the risk estimates were described as being developed in the context of a specific decision. Surprisingly, despite the early recognition of the importance of uncertainty in RBDM mentioned earlier, only 52% of the publications included any significant discussion of the use of uncertainty estimates in the decision.

### 3.3 Themes within RBDM

Building on the foundational characteristics, three themes emerged as additional ways that RBDM could be defined. Those themes are: (1) risk acceptability; (2) prioritization; and (3) stakeholder engagement. The list of publications and themes each represents is provided in Appendix A.

The first theme in RBDM was risk acceptability and the recognition of the need to consider the tradeoffs between risk and other factors. Twenty‐four of the 61 publications (39%) included risk acceptability in their discussion of RBDM. These publications discuss that there are factors besides risk that may need to be accounted for in the decision‐making process. For example, Barlow et al. (2015) discuss the need to consider “acceptability of risk” in the decision. The National Research Council Committee on Best Practices for Risk‐Informed Remedy Selection, Closure and Post‐Closure of Contaminated Sites states “the decision‐making process requires the balancing of risk against benefit” (2014). In these publications, risk acceptability is operationalized in several ways, including (1) an explicit recognition that zero risk is not the goal (Delage et al., 2019); (2) a need to make tradeoffs with factors besides risk, such as cost (Latinopoulos, Mylopoulos, & Mylopoulos, 1997; Li, Huang, & Xue, 2014; Manap & Voulvoulis, 2014); and (3) discussion of the risk appetite or risk tolerance of the decisionmakers (Leach Bennett et al., 2011; Lipton & Gillett, 1992; Mirabi, Mianabadi, Zarghami, Sharifi, & Mostert, 2014; Opitz‐Stapleton et al., 2019). This theme is the one that is most aligned with the decision analysis modeling tool, multicriteria decision analysis. In this theme, RBDM encourages the decisionmaker to move away from setting standards based only on estimates of risk and adds discussion of using tradeoffs with other factors or risk tolerance as a means of informing the decision.

#### 3.3.1 Prioritization

The second theme found in the literature is that RBDM is about priority setting; that is, about comparing one risk or one risk management intervention to another. In these cases, decisions are based on allocation of limited resources such as remediation funds for leaking underground storage tanks (Connor & McHugh, 2002), resources to manage aging urban water infrastructure (Mamo, 2015), or time in the operating room (Abbasgholizadeh Rahimi, Jamshidi, Ait‐Kadi, & Bartolome, 2016). This theme could be considered a subset of risk acceptability in that is forces the consideration of at least one factor in addition to risk—one related to resources. Twelve of the 61 publications (20%) connected RBDM with prioritization. Several authors used information on risk, along with other factors, to decide how to rank or prioritize risk management activities. Haimes and Stakhiv discuss using RBDM to accomplish the “proper allocation of resources” (Haimes & Stakhiv, 1986). Jenni, Merkhofer, and Williams (1995) review the “risk‐based priority setting” that was developed to inform decisions about how to allocate an environmental restoration budget across multiple sites. Importantly, prioritization (i.e., public health risk ranking) was a key step in the RBDM approach recommended by the National Academies Committee for food safety (Institute of Medicine & National Research Council, 2010).

#### 3.3.2 Stakeholder Engagement

The third theme within RBDM that was identified in the analysis is the use of RBDM to more effectively engage stakeholders in the decision‐making process. Fourteen of the 61 publications (23%) included “stakeholder engagement” or “public engagement” in their definition of RBDM. The way that stakeholders were included varied across these publications with some involved in problem definition, some in providing data and information to estimate risks and other factors, and others in providing input on their assessment of risk acceptability. For example, in one paper, stakeholders provided input about what was important for the problem relative to assessment of risk for national parks (Carey, Beilin, Boxshall, Burgman, & Flander, 2007). Another paper discussed increasing inclusion of stakeholder knowledge and preferences (Hart et al., 2006). Hart et al. (2006) also discussed how the risk assessment process can alienate stakeholders, so creating connections for stakeholders to understand and engage is important. Jenni et al. (1995) provides a cautionary tale about what can happen when stakeholders are not fully engaged in the decision‐making process; a large investment in analytical tools to support RBDM was shelved because of lack of public engagement. Naime and Andrey identify public participation as one of the 13 principles of risk‐based regulation (Naime & Andrey, 2013). Sexton offers a review of public participation in the assessment and management of environmental health risks in the United States, and states that “meaningful public engagement is an inherent component of all phases of the risk assessment‐risk management paradigm” (Sexton, 2013). Wolt and Peterson state that the involvement of stakeholders is “pivotal to the successful application of risk assessment in agricultural biotechnology” (Wolt & Peterson, 2000). All 14 publications considered stakeholder engagement a core tenet of a risk‐based approach.

#### 3.3.3 Intersection of Themes

**Fig 4:**
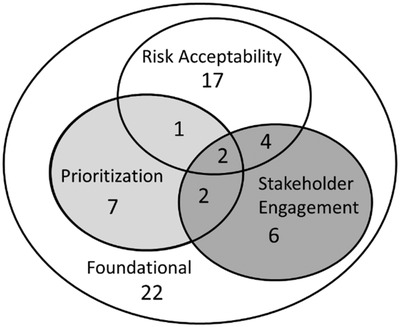
Publication counts by theme

Several of the 61 papers included in this review included multiple themes in their definition of RBDM (Fig. 4). The most common theme was risk acceptability, with prioritization and stakeholder engagement relatively evenly represented. There were only two publications that included all three themes and both were National Academy Committee reports with broad scopes (Institute of Medicine & National Research Council, 2010; National Research Council (US) and Institute of Medicine (US) Committee on Ranking FDA Product Categories Based on Health Consequences, Phase II, 2011). Four publications included both the concepts of risk acceptability and stakeholder engagement. Three of the four focused on blood safety and were based on the same RBDM framework for blood safety (Leach Bennett et al., 2011; O'Brien et al., 2019; Stein et al., 2011). The remaining paper focused on food safety (Barlow et al., 2015). Only one publication included both risk acceptability and prioritization in their definition of RBDM (Haimes & Stakhiv, 1986). Finally, two publications included both prioritization and stakeholder engagement in their definition of RBDM (Jenni et al., 1995; Pasquier et al., 2020).

### 3.4 Definition of RBDM

The intent of finding one common definition of RBDM across these publications evolved into the development of a multifaceted definition. In addition to the two observed commonalities among these publications, a third component was added to the foundational definition based on normative expectations from the cited National Academies studies and other sources about the key role that uncertainty plays in RBDM. Therefore, we propose the following foundational definition: RBDM is an approach that (1) utilizes estimates of the likelihood and severity of harm (i.e., a risk estimate) to inform decisions; (2) aligns risk estimates with the needs of decisionmakers, and (3) includes explicit consideration of uncertainty in the risk estimates. Once a process meets this foundational definition, there are three extensions to the definition that could be considered: risk acceptability, prioritization, and stakeholder engagement. These extensions should be considered relative to the decision context and decision needs. Note that there is no specification of quantitative risk estimates or uncertainty estimates; rather, qualitative consideration of risk and uncertainty may be a reasonable first step. In fact, when moving into the structured use of risk information to inform decisions, some regulatory organizations find that a qualitative approach is more acceptable as a starting place (US FDA, 2018).

**Fig 5:**
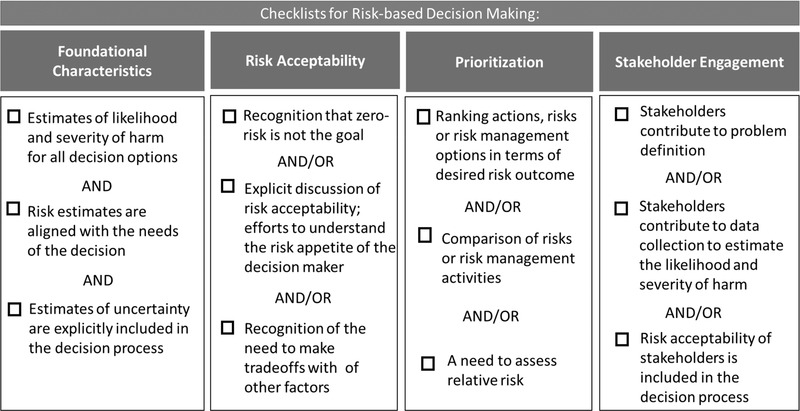
Checklist of criteria for RBDM by theme

To aid in the development and evaluation of RBDM processes, a checklist of the characteristics of a RBDM process for the foundational definition, as well as a description of what would be included for each of the three themes was also developed (Fig. 5). This checklist could be used *a priori* as a planning tool or post hoc to guide an evaluation.

## 4 DISCUSSION

This scoping review identified several important findings that can help improve terminology and language around the use of RBDM. Specifically, this review found that the inclusion of estimates of likelihood and severity of harm that are aligned with decision needs is common across all identified publications. Therefore, these two components form our foundational definition for RBDM and can help clarify guidance and expectations around the use of RBDM.

The breadth of topic areas and journals in which publications were found indicate that RBDM is relevant for a wide range of application areas. It is important to note that this reflects only a fraction of the diversity of applications for RBDM as 98% of the publications identified in the search were excluded from this review, suggesting that RBDM is a commonly used term but is not regularly defined. The wide use of RBDM makes convening on a single definition challenging. It also highlights the difficulty a researcher would have in developing a comprehensive definition based only on their knowledge of the literature.

The large number of publications in the topic areas of environmental science and engineering and infrastructure provides evidence that these fields may be most experienced with this approach. However, these results should not be over‐interpreted, as they are, to some degree, an artifact of the journals that were part of the catalogs used for this scoping review. Both of topic areas had publications in *Risk Analysis*, but there was a higher concentration of environmental science papers in one journal (*Science of the Environment*). The engineering and infrastructure papers were spread across more journals as well as book chapters and conference proceedings, suggesting there may be a stronger connection of thought in the environmental science field than in engineering and infrastructure.

The connection between the analysis and the decisionmaker was a foundational component of RBDM with several papers emphasizing the need for risk estimates to be aligned with the needs of the decisionmaker. Robinson and Levy (Robinson & Levy, 2011) amplify the recommendations from the 2008 National Academy report to the Environmental Protection Agency (National Research Council, 2008) by discussing the need for an iterative and integrated process framed in terms of the specific decision context and designed to meet the objectives of the decisionmaker. Similarly, Ahmadisharaf and Benham propose the use of a Bayesian framework for helping the decisionmakers to understand the risks (Ahmadisharaf & Benham, 2020). Finally, both Bell (2019) and Carriger and Newman (Carriger & Newman, 2012) discuss the value of influence diagrams to connect the analysis to the decision.

One critical aspect of RBDM that was missing in much of the identified literature is the need to incorporate uncertainty in the risk estimates into the decision‐making process. From a decision analysis perspective, one of the main reasons that RBDM is difficult is the uncertainty in the estimates. In fact, the need to rigorously address uncertainty has previously been identified as one of the main components of RBDM (Peterman & Anderson, 1999). However, almost half of the reviewed publications did not mention the need to incorporate uncertainty into the decision‐making process. This is a significant gap since much adequately incorporating uncertainty into RBDM is challenging and guidance is needed. Therefore, we propose that uncertainty analysis be added to the foundational definition, even though it was not a finding from the literature.

Our proposed foundational definition for RBDM may seem too basic for those who have used the term for many years and have a more advanced understanding of what RBDM means. However, this foundational definition provides a starting point for what is needed to develop a decision‐making system that is risk‐based, particularly in fields where RBDM is an emerging approach. For example, countries that are just beginning to establish systems for improving environmental or food safety systems often start with a hazard‐based approach to make decisions, which can lead to an investment of resources that do not result in a meaningful reduction of risk. The initial stages of addressing a food safety issue (or building a food safety system) may be triggered by a specific event that received significant public attention. In that case, risk aversion from the risk manager may require quick action, and a hazard‐based approach will be quicker than a risk‐based one. Emphasizing the value of basing decisions on estimates of the likelihood and severity of harm can lead to more effective and efficient risk reduction in the long run. Our proposed definition can increase awareness for the need to develop risk estimates and work with decisionmakers and, thus, build capacity for moving beyond a hazard‐based approach. For the development of a RBDM system, building the data and analytical capacity to estimate risk and to align the analysis with the needs of the decision are key steps to building a RBDM system.

A fundamental question about RBDM is whether and how it is distinct from risk management, which is generally defined to be the process of identifying, assessing, and controlling threats. In *Science and Decisions: Advancing Risk Assessment* (National Research Council, 2008), the National Research Council emphasizes the importance of conducting risk assessments for the purpose of risk management; that is, using estimates of the likelihood and severity of harm to inform the decision. In that vein, some may use the terms “risk‐based decision making” and “risk management” interchangeably. However, the general use of the term risk management includes any action to address a risk, which may not include an explicit estimate of risk, whereas RBDM requires that the decision to act is informed by consideration of the likelihood and severity of harm. In other words, risk management can and does include actions taken when there are no estimates of risk generated. For example, decisions that use default safety factors or other “bright lines” would qualify as risk management decisions, but not as RBDM. Therefore, risk management is a broader term than RBDM; to say it another way, every risk‐based decision is a risk management decision, but every risk management decision is not a risk‐based decision.

Those who are interested in applying RBDM may appreciate the flexibility of using the theme(s) that are most aligned with their needs. Inclusion of the themes in decisions should be taken on incrementally and should be based on the needs of the decisionmaker. Incorporating risk acceptability, for example, may be required or prohibited by law or statute for some decisions. If not required or prohibited, the nature of the decision should be considered to determine if this theme of RBDM is appropriate. Similarly, if the decision has to do with allocating scarce resources, the components of the prioritization theme are relevant. And finally, for decisions that will impact stakeholders, the components of the stakeholder engagement theme of the definition should be considered.

There are two main limitations to this work. First, since the goal of this work was to develop one clear definition for RBDM, it was necessary to use “RBDM” as part of the search strategy. This likely resulted in publications that have the characteristics described in the foundational definition and the three theme definitions being excluded from this scoping review. For example, a 2018 literature review identified 253 publications that used public health information to rank food safety risks (Van der Fels‐Klerx et al., 2018). Many of the publications identified in that review did not specifically use the term “RBDM,” so they were not included in this scoping review even though they likely met the foundational and prioritization definitions. Similarly, there is a large amount of literature on comparative risk assessment, which began in the 1980s with “Unfinished Business: A Comparative Assessment of Environmental Problems” (U.S. Environmental Protection Agency, 1987), that fall in the realm of RBDM but were not included in this review. For example, Caswell (2008) focused on multifactorial risk prioritization but did not specifically reference RBDM and, as such, was not identified in the search. The breadth of search to use should be considered in future systematic reviews on this topic. Second, this review focused on literature relevant for food safety and, as such, the search strategy was limited to agricultural, environmental, and medical catalogs even though RBDM is used in many other areas. For example, only one publication on security and defense was identified even though RBDM is used broadly in that field (Hamilton, Hong, Casman, & Gurian, 2015). Even with these limitations, the collection of articles is quite broad and lends weight to the use of the definition derived from this review.

## 5 CONCLUSION

The idea that RBDM is a valuable goal to work toward is well developed across many fields of human and ecological risk. This research develops, for the first time, a set of literature‐driven characteristics that can be used by those who are aiming to implement risk‐based approaches. Having one clear definition for RBDM will support the consistent use of the term across disciplines and, therefore, increase the pace of learning and adoption in the various fields RBDM is used. Further, as work on food safety systems with LMIC advances, the proposed checklist will provide a critical reference point that can be used to direct those who are interested in moving toward a risk‐based food safety system. With this clear definition, RBDM can shift from an aspirational goal to an achievable plan.

## Appendix A Publications in Scoping Review

### A.1

**Table:**

Reference	Year	Topic	Theme
Abbasgholizadeh Rahimi et al. (2016)	2016	Medical	Prioritization
Ahmadisharaf and Benham (2020)	2020	Environmental Science	Foundational
Barlow et al. (2015)	2015	Food Safety	Risk Acceptability and Stakeholder Engagement
Bell (2019)	2019	Engineering and infrastructure	Foundational
Bereskie, T., Haider, H., Rodriguez, M. J., & Sadiq, R. (2017). Small drinking water systems under spatiotemporal water quality variability: A risk‐based performance benchmarking framework. *Environmental Monitoring and Assessment*, *189*(9), 464. https://doi.org/10.1007/s10661‐017‐6176‐z.	2017	Engineering and Infrastructure	Foundational
Blacker, S., & Goodman, D. (1994). Risk‐based decision making: Case study: Application at a superfund cleanup. *Environmental science and Technology*, *28*(11). https://doi.org/10.1021/es00060a002	1994	Environmental science	Foundational
Carey et al. (2007)	2007	Environmental science	Stakeholder Engagement
Carriger and Newman (2012)	2012	Environmental science	Foundational
Committee on Best Practices for Risk‐Informed Remedy Selection, Closure and Post‐Closure of Contaminated Sites, Nuclear and Radiation Studies Board, Division on Earth and Life Studies, Science and Technology for Sustainability Program, Policy and Global Affairs, & National Research Council (2014)	2014	Environmental science	Risk acceptability
Connor and McHugh (2002)	2002	Environmental science	Prioritization
Davies, L. P. (2002). Risk assessment in the UK nuclear power industry. *Safety Science*, *40*(1–4): 203–230. https://doi.org/10.1016/S0925‐7535(01)00037‐6.	2002	Engineering and Infrastructure	Foundational
Delage et al. (2019)	2019	Medical	Risk Acceptability
Fletcher, Warrick (Rick) J. (2015). Review and refinement of an existing qualitative risk assessment method for application within an ecosystem‐based management framework. *ICES Journal of Marine Science/Journal Du Conseil*, *72*(3), 1043–1056. https://doi.org/10.1093/icesjms/fsu142.	2015	Ecological	Foundational
Grafton Q., McLindin M., Hussey K., Wyrwoll P., Wichelns D., Ringler C., … Williams, J. (2016). Responding to global challenges in food, energy, environment and water: Risks and options assessment for decision‐making. *Asia and the Pacific Policy Studies*, *3*(2), 275–299. https://doi.org/10.1002/app5.128.	2016	Natural disasters	Foundational
Güngör‐Demirci, G., Lee, J., Keck, J., Harrison, S. J., & Bates, G. (2019). Development of a risk‐based tool for groundwater well rehabilitation and replacement decisions. *Journal of Water Supply: Research and Technology – Aqua*, *68*(6), 411–419.	2019	Engineering and Infrastructure	Prioritization
Haimes and Stakhiv (1986)	1986	Engineering and Infrastructure	Risk Acceptability and Prioritization
Hamilton et al. (2015)	2015	Security and defense	Foundational
Han, P. K. J., Hootsmans, N., Neilson, M., Roy, B., Kungel, T., Gutheil, C., Diefenbach, M., & Hansen, M. (2013). The value of personalised risk information: A qualitative study of the perceptions of patients with prostate cancer. *BMJ Open*, *3*(9), e003226. https://doi.org/10.1136/bmjopen‐2013‐003226	2013	Medical	Foundational
Hart et al. (2006)	2006	Ecological	Stakeholder Engagement

Reference	Year	Topic	Theme
Institute of Medicine & National Research Council (2010)	2010	Food safety	All
Jenni et al. (1995)	1995	Environmental science	Prioritization and Stakeholder Engagement
Kim, D., & Kaluarachchi, J. J. (2016). A risk‐based hydro‐economic analysis for land and water management in water deficit and salinity affected farming regions. *Agricultural Water Management*, *166*, 111–122. http://www.sciencedirect.com/science/publication/pii/S0378377415301852.	2016	Environmental science	Prioritization
Kirchsteiger, C. (2002). International workshop on promotion of technical harmonisation on risk‐based decision‐making. *Safety Science*, *40*(1–4), 1–15. https://doi.org/10.1016/S0925‐7535(01)00033‐9.	2002	Regulatory	Foundational
Kirchsteiger, C., & Cojazzi, G. (2000). Promotion of technical harmonisation on risk‐based decision making. In S. Kondo and K. Furuta (Eds.), *Psam 5: Probabilistic safety assessment and management (*Vols. *1–4*, pp. 1497–1504). Tokyo: Universal Academy Press.	2002	Regulatory	Foundational
Koutsoumanis, K. P., & Aspridou, Z. (2016). Moving towards a risk‐based food safety management. *Current Opinion in Food Science*, *12*, 36–41. https://doi.org/10.1016/j.cofs.2016.06.008.	2016	Food Safety	Foundational
Latinopoulos et al. (1997)	1994	Engineering and Infrastructure	Risk Acceptability
Leach Bennett et al. (2011)	2011	Medical	Risk Acceptability and Stakeholder Engagement
Li et al. (2014)	2014	Engineering and Infrastructure	Risk Acceptability
Lipton and Gillett (1992)	1992	Environmental science	Risk Acceptability
Mamo (2015)	2015	Engineering and Infrastructure	Prioritization
Manap and Voulvoulis (2014)	2014	Ecological	Risk Acceptability
McCloskey, B., Zumla, A., Lim, P. L., Endericks, T., Arbon, P, Cicero, A., & Borodina, M. (2020). A risk‐based approach is best for decision making on holding mass gathering events. *Lancet (London, England)*, *395*(10232), 1256–1257. https://doi.org/10.1016/S0140‐6736(20)30794‐7.	2020	Public Health	Foundational
Mengersen, K., & Whittle, P. (2011). Improving accuracy and intelligibility of decisions. *Journal Für Verbraucherschutz Und Lebensmittelsicherheit*, *6*(Supplement 1), S15–19. https://doi.org/10.1007/s00003‐011‐0694‐3.	2011	Agriculture	Foundational
Menitove, J. E., Leach Bennett, J., Tomasulo, P., & Katz, L. M. (2014). How safe is safe enough, who decides and how? From a zero‐risk paradigm to risk‐based decision making. *Transfusion*, *54*(3 Pt 2), 753–757. https://doi.org/10.1111/trf.12569.	2014	Medical	Risk Acceptability
Michalopoulos, E., Georgiou, A. C., & Paparrizos, K., (2008). Risk‐based decision making and risk management of European union regional programs. *Yugoslav Journal of Operations Research*, *18*(1), 75–94. https://doi.org/10.2298/YJOR0801075M	2008	Regulatory	Foundational
Mirabi et al. (2014)	2014	Engineering and Infrastructure	Risk Acceptability
Motahari, A., Malmasi, S., & Haghighi Fard, N. Jafarzahe. (2015). Application of a multi‐criteria decision‐making approach for ranking environmental risks caused by petrochemical industries: A case study of a sodium carbonate production unit. *Human and Ecological Risk Assessment*, *21*, 358–374 https://doi.org/10.1080/10807039.2014.916548.	2015	Environmental science	Prioritization
Naime and Andrey (2013)	2013	Regulatory	Stakeholder Engagement
National Research Council (US) and Institute of Medicine (US) Committee on Ranking FDA Product Categories Based on Health Consequences, Phase II (2011)	2011	Food safety	All

Reference	Year	Topic	Theme
O'Brien et al. (2019)	2019	Medical	Risk Acceptability and Stakeholder Engagement
Opitz‐Stapleton et al. (2019)	2019	International development	Risk Acceptability
Pan, J., Oates, C. J., Ihlenfeld, C., Plant, J. A., & Voulvoulis, N. (2010). Screening and prioritisation of chemical risks from metal mining operations, identifying exposure media of concern. *Environmental Monitoring and Assessment*, *163*(1–4), 555–571.	2010	Environmental science	Prioritization
Pasquier et al. (2020)	2020	Natural disasters	Prioritization and Stakeholder Engagement
Philipson, L. L. (1983). Risk acceptance criteria and their development. *Journal of Medical Systems*, *7*(5), 437–456.	1983	Medical	Risk Acceptability
Pollard, S. J., Davies, G. J., Coley, F. H., & Lemon, M. (2008). Better environmental decision making–Recent progress and future trends. *Science of the Total Environment*, *400*(1/3), 20–31. https://doi.org/10.1016/j.scitotenv.2008.07.022.	2008	Environmental science	Foundational
Ragas, A. M. J., Huijbregts, M. A. J., Henning‐de Jong, I., & Leuven, R. S. E. W. (2009). Uncertainty in environmental risk assessment: Implications for risk‐based management of river basins. *Integrated Environmental Assessment and Management (IEAM) 5*(1), 27–37. https://doi.org/10.1897/IEAM_2008‐046.1.	2009	Environmental science	Foundational
Rehan, B. M. (2018). accounting public and individual flood protection measures in damage assessment: A novel approach for quantitative assessment of vulnerability and flood risk associated with local engineering adaptation options. *Journal of Hydrology*, *563*, 863–873. https://doi.org/10.1016/j.jhydrol.2018.06.061.	2018	Natural disasters	Risk Acceptability
Robinson and Levy (2011)	2011	Regulatory	Foundational
Sabbaghian, R. J., M. Zarghami, M., Nejadhashemi, A. P., Sharifi, M. B., Herman, M. R., & Daneshvar, F. (2016). Application of risk‐based multiple criteria decision analysis for selection of the best agricultural scenario for effective watershed management. *Journal of Environmental Management*, 168, 260–272. http://www.sciencedirect.com/science/journal/03014797.	2016	Agriculture	Risk Acceptability
Sadiq, R., Husain, T., Veitch, B., & Bose, N. (2004). Risk‐based decision‐making for drilling waste discharges using a fuzzy synthetic evaluation technique. *Ocean Engineering*, *31*(16), 1929–1953. https://doi.org/10.1016/j.oceaneng.2004.05.001	2004	Environmental science	Risk Acceptability
Sam, K., Coulon, F., & Prpich, G. (2017). A multi‐attribute methodology for the prioritisation of oil contaminated sites in the Niger delta. *Science of the Total Environment*, *579*, 1323–32. https://doi.org/10.1016/j.scitotenv.2016.11.126.	2017	Environmental science	Prioritization
Sexton (2013)	2013	Environmental science	Stakeholder Engagement
Stein et al. (2011)	2011	Medical	Risk Acceptability and Stakeholder Engagement
Styles, C. E., Seed, C. R., Hoad, V. C., Gaudieri, S., & Keller, A. J. (2017). Reconsideration of blood donation testing strategy for human T‐cell lymphotropic virus in Australia. *Vox Sanguinis*, *112*(8), 723–732. https://doi.org/10.1111/vox.12597.	2017	Medical	Risk Acceptability
Umber, J., Culhane, M., Cardona, C., & Goldsmith, T. (2019) A risk‐based permitting process for the managed movement of animals and products of animal origin as a tool for disease management. *Frontiers in Veterinary Science*, *6*, 433. https://doi.org/10.3389/fvets.2019.00433.	2019	Agriculture	Foundational
Wang, J., & Yuan, H. (2011). Factors affecting contractors’ risk attitudes in construction projects: Case study from China. *International Journal of Project Management*, *29*(2), 209–219. https://doi.org/10.1016/j.ijproman.2010.02.006.	2011	Engineering and Infrastructure	Risk Acceptability

Reference	Year	Topic	Theme
Wang, Z. Z., & Zhang, Y. X. (2011). Risk based decision making for bridge under multi hazard. *Applied Mechanics and Materials*, *105–107*, 1215–1219. https://doi.org/10.4028/www.scientific.net/AMM.105‐107.1215.	2012	Engineering and Infrastructure	Risk Acceptability
Willoughby, A., Andreassen, P. R., & Toland, A. E. (2019). Genetic testing to guide risk‐stratified screens for breast cancer. *Journal of Personalized Medicine*, *9*(1). https://doi.org/10.3390/jpm9010015	2019	Medical	Foundational
Wolt and Peterson (2000)	2000	Agriculture	Stakeholder Engagement
Xenidis, Y., & Angelides, D. C. (2013). *A risk‐based decision making methodology for planning and operating safe infrastructure systems against various hazards*. Vol. 30. Computational Methods in Applied Sciences. Springer Netherland. https://doi.org/10.1007/978‐94‐007‐6573‐3_27.	2013	Engineering and Infrastructure	Risk Acceptability
Younger, P. L., Coulton, R. H., & Froggatt, E. C. (2005). The contribution of science to risk‐based decision‐making: Lessons from the development of full‐scale treatment measures for acidic mine waters at Wheal Jane, UK. *Science of the Total Environment*, *338*(1/2), 137–54. https://doi.org/10.1016/j.scitotenv.2004.09.014.	2005	Environmental science	Stakeholder Engagement
